# Successful use of whole genome amplified DNA from multiple source types for high-density Illumina SNP microarrays

**DOI:** 10.1186/s12864-018-4572-6

**Published:** 2018-03-06

**Authors:** Casey L. Dagnall, Lindsay M. Morton, Belynda D. Hicks, Shengchao Li, Weiyin Zhou, Eric Karlins, Kedest Teshome, Salma Chowdhury, Kerrie S. Lashley, Joshua N. Sampson, Leslie L. Robison, Gregory T. Armstrong, Smita Bhatia, Gretchen A. Radloff, Stella M. Davies, Margaret A. Tucker, Meredith Yeager, Stephen J. Chanock

**Affiliations:** 1 Division of Cancer Epidemiology and Genetics, National Cancer Institute (NCI), National Institutes of Health (NIH), Rockville, MD USA; 2 Cancer Genomics Research Laboratory, Leidos Biomedical Research, Inc., Frederick National Laboratory for Cancer Research, Frederick, MD USA; 3 Department of Epidemiology and Cancer Control, St. Jude Children’s Research Hospital, Memphis, TN USA; 4 Department of Population Sciences, City of Hope, Duarte, CA USA; 5 Institute for Cancer Outcomes and Survivorship, School of Medicine, University of Alabama at Birmingham, Birmingham, AL USA; 6 Department of Pediatrics, Cincinnati Children’s Hospital Medical Center, Cincinnati, OH USA

**Keywords:** Whole genome amplification, Genome-wide association study, High-density microarray, CCSS clinical trial

## Abstract

**Background:**

The recommended genomic DNA input requirements for whole genome single nucleotide polymorphism microarrays can limit the scope of molecular epidemiological studies. We performed a large-scale evaluation of whole genome amplified DNA as input into high-density, whole-genome Illumina® Infinium® SNP microarray.

**Results:**

Overall, 6622 DNA samples from 5970 individuals were obtained from three distinct biospecimen sources and genotyped using gDNA and/or wgaDNA inputs. When genotypes from the same individual were compared with standard, native gDNA input amount, we observed 99.94% mean concordance with wgaDNA input.

**Conclusions:**

Our results demonstrate that carefully conducted studies with wgaDNA inputs can yield high-quality genotyping results. These findings should enable investigators to consider expansion of ongoing studies using high-density SNP microarrays, currently challenged by small amounts of available DNA.

**Electronic supplementary material:**

The online version of this article (10.1186/s12864-018-4572-6) contains supplementary material, which is available to authorized users.

## Background

Genome-wide association studies (GWAS) performed in the past decade have been instrumental in advancing the discovery of genetic variants associated with many human diseases and traits [1]. Careful study design is critical for robust findings, and particularly should include sufficient sample size (e.g. number of cases and controls) to ensure adequate power to detect genetic associations [2]. Oftentimes large sample sizes are necessary, especially in GWAS of rare variants as well as for the discovery of common variants with small estimated odds ratios (< 1.05). Additional key considerations include disease prevalence, linkage disequilibrium, and the estimated effect size of the genetic variants, which is frequently inversely related with allele frequency [3]. Commercial single nucleotide polymorphism (SNP) microarrays, which have emerged as the standard for GWAS, require a standard amount of input DNA. These DNA template requirements can be high enough to result in the exclusion of a sizeable fraction of desired samples due to insufficient amounts of available DNA, which in turn can compromise the statistical power of a GWAS.

Whole genome amplification (WGA) techniques can increase the amount of DNA template, eliminating sample exclusions from a GWAS due to limited DNA availability. While success has been observed using whole genome amplified DNA (wgaDNA) as a suitable alternative to genomic DNA (gDNA) for lower-density microarrays [4–7], there has been no large methodological study published regarding the efficiency of wgaDNA using the newer high-density microarrays that include large numbers of rare or uncommon variants. Though it is an option to improve sample inclusion, many investigators have avoided using wgaDNA because of the potential for biased amplification due to PCR-based methods of WGA. The multiple displacement amplification (MDA) method for WGA uses φ29 DNA polymerase to perform isothermal amplification, in which non-specific binding of random hexamers to denatured gDNA is followed by strand displacement synthesis at a constant temperature, allowing highly uniform amplification across the genome due to the high processivity and fidelity of the φ29 DNA polymerase [8–11]. Input of wgaDNA has been reported to result in a decreased call rates, thought to be related to WGA methods that could yield allelic biases [12].

One of the commercial arrays with a high density of both common and uncommon SNPs is the HumanOmni5Exome BeadChip, which contains more than 4.3 million tag SNPs selected from the International HapMap and 1000 Genomes Projects [13, 14], as well as more than 240,000 functional exonic variants, most of which are rare or uncommon. The commercial recommendation for the HumanOmni5Exome BeadChip requires a starting genomic DNA (gDNA) template amount of at least 400 ng (8 μL at 50 ng/μL). Using the recommended automated Infinium® assay protocols, designed by Illumina® for the Tecan eight-tip robots, a minimum of 30 μL of starting material (approximately 1500 ng of gDNA per sample) is recommended to accurately transfer the 8 μL of sample into the amplification procedure of the assay, even though less than a third is used. The requirement of 1500 ng of gDNA often results in many samples intended for a GWAS failing to qualify for inclusion in the study, thereby compromising its power. Notably, the Infinium® Assay already includes, as a first step, a proprietary whole-genome amplification, using a version of the MDA method, to increase the quantity of input DNA by several thousand-fold without introducing substantive amplification bias [15].

We conducted a large-scale study of 6258 samples from 5970 individuals (3932 gDNA and 2326 wgaDNA samples; Table 1) to investigate whether the performance of wgaDNA as input to the Infinium technology is comparable to that of gDNA. Samples were derived from the Childhood Cancer Survivor Study, a multi-center, long-term follow-up study that collected germline DNA with whole blood samples, Oragene and mouthwash buccal samples [16]. We evaluated the performance of DNA from various source types and their effectiveness on the HumanOmni5Exome BeadChip when using wgaDNA versus gDNA. Blinded quality control (QC) replicates were interspersed across plates, including 81 individuals as full QC replicates (2 gDNA and 2 wgaDNA samples) and another 44 as partial QC replicates (*n* = 21, 1 gDNA and 1 wgaDNA sample; *n* = 18, 2 gDNA samples; *n* = 5, 2 wgaDNA samples). The fidelity of wgaDNA as an input was assessed by the following metrics: concordance of SNP calls, completion rates per samples and per locus differences between the gDNA and wgaDNA inputs.

**Table 1:** Samples and source material utilized for alternate input testing

Sample Type	Source Material	Total
gDNA		3932
	Blood	745
	Buccal	1057
	Oragene	2130
wgaDNA		2326
	Blood	602
	Buccal	727
	Oragene	997
Total Samples		6258

## Results

### Whole genome amplification

Based on completion and concordance rates between gDNA and wgaDNA generated by different WGA techniques in a previous study [17], we used the GE Healthcare’s illustra™ GenomiPhi™ V2 DNA Amplification Kit, based on the MDA technology with φ29 DNA polymerase [18]. WGA was attempted for 2548 samples, of which a fraction (*n* = 179) included multiple attempts on the same individual samples. We obtained average yields of 5.1 μg of amplified DNA, with fragment sizes typically greater than 13 Kb. These values reflect the quality of the starting gDNA; we observed that the input wgaDNA should be of an average size of at least 2 kb for optimal genotyping with Infinium® microarrays [12]. We used the AmpF*l*STR® Identifiler® DNA profiling assay as an additional quality check to identify wgaDNA samples for contamination, poor wgaDNA quality, or poor amplification quality [11, 19–21]. 491 wgaDNA samples generated were excluded from genotyping (*n* = 454 did not yield 1500 ng for input; *n* = 37 contaminated or discordance with reported gender). No wgaDNA samples were excluded due to amplification quality or allelic drop-out or allelic imbalance introduced by WGA, but they were evaluated in analyses that follow.

### Sample completion & heterozygosity

Of the 4,511,679 designed and manufactured probes on the array, 8512 failed completely (no genotypes from gDNA or wgaDNA) and an additional 50,816 probes failed across all wgaDNA samples. Call rates of individual samples were calculated by dividing the number of informative probes per sample by the number of non-missing probes by sample type (4,503,167 for gDNA; 4,452,351 for wgaDNA). Ten samples (6 gDNA, 4 wgaDNA) failed to load into the GenomeStudio™ software and were excluded from further analysis. 95 samples (gDNA) were also excluded from further analysis due to a systematic error during processing which was isolated to one source plate, which included 5 QC replicates. Call rates by chip were evaluated (Additional file 1: Table S1) and identified 8 chips, in which all 4 samples had low call rates, indicating systematic error. The 32 samples (wgaDNA = 16; gDNA = 16) were excluded from further analysis. Half of the chips (*n* = 8) with 3 sample failures (out of 4 samples on the chip) were from common source plates containing wgaDNA samples, suggesting a problem during processing but since this could not be confirmed, these samples (*n* = 32) were retained for the analysis. Results were similar when we repeated analyses excluding these 32 samples (not shown).

**Fig. 1:**
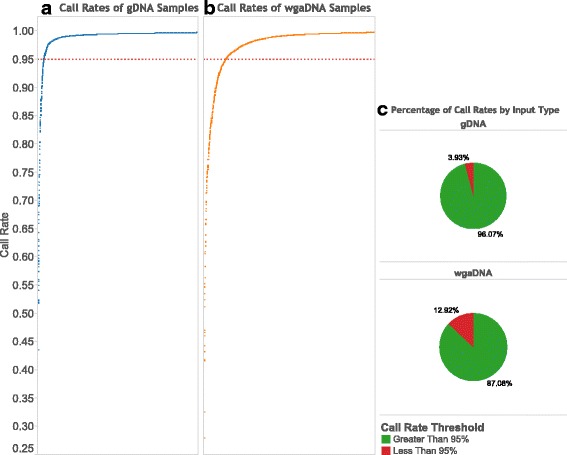
Call rates of samples by input type. **a**) Call rates of gDNA samples (*n* = 3818) and **b**) wgaDNA samples (*n* = 2306), where the dotted line displays the call rate threshold of 95%. **c**) Percentage of call rates above and below 95% threshold for each input type

The percentage of samples with a call rate less than 95% was 7.32% (*n* = 448); however, the failure rate was higher for wgaDNA, 12.92% (*n* = 298) samples than gDNA, 3.93% (*n* = 150) samples (Fig. 1). We compared the call rates of QC replicates and found no correlation within or between input types (Additional file 1: Figure S1).

**Fig. 2:**
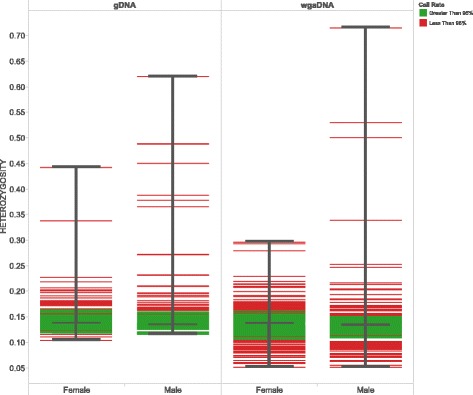
Heterozygosity rates of samples (*n* = 6124) by input type (gDNA, wgaDNA) and gender. Color depicts samples that fall above (green) or below (red) the call rate threshold of 95%

The distribution of heterozygosity rates had an overall median of 0.137 (upper and lower quartiles of 0.138 and 0.135; range 0.052 to 0.716). The majority of the outliers (*n* = 220) were samples with call rates less than 95%, which can be an indication of poor performance during quality control assessment. However, a small subset of samples (*n* = 46) were outliers but had call rates greater than 95%. Median heterozygosity rates did not differ between gDNA and wgaDNA input types (Fig. 2).

### Sample source & quality

**Fig. 3:**
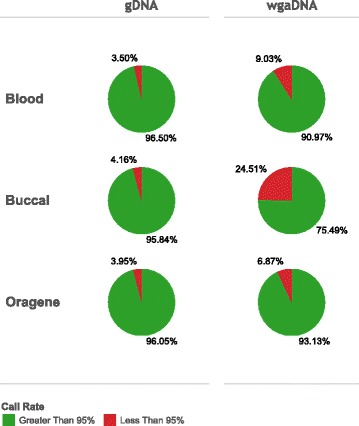
Call rates of samples by input type and source material: gDNA (*n* = 685 Blood; *n* = 1034 Buccal; *n* = 2099 Oragene), wgaDNA (*n* = 598 Blood; *n* = 718 Buccal; *n* = 990 Oragene)

We also considered source material (blood, buccal, Oragene) and differences between gDNA and wgaDNA inputs (Fig. 3). Sample failure, based on call rates of gDNA samples across the three source materials, were comparable: blood (3.50%), buccal (4.16%), and Oragene (3.95%). wgaDNA sample failure, based on sample call rates across the three source materials, differed by source and were higher in buccal (24.51%) compared with blood (9.03%) and Oragene (6.87%).

Based on Identifiler assay results, we categorized quality based on the number of markers; this approach evaluated alleles amplified and the number of markers in wgaDNA samples, which displayed allele drop-out, which could indicate potential loss of heterozygosity genotypes. If poor quality wgaDNA samples were excluded initially from being run on chips, this would have reduced the overall wgaDNA sample failure rate to 7.24%, with failure rates reduced in buccal (13.69%), blood (5.42%), and Oragene (4.36%) samples. This quality measure suggests that WGA of buccal samples may have a higher failure rate than the other source materials (Additional file 1: Figure S2).

### Genotype concordance

**Fig. 4:**
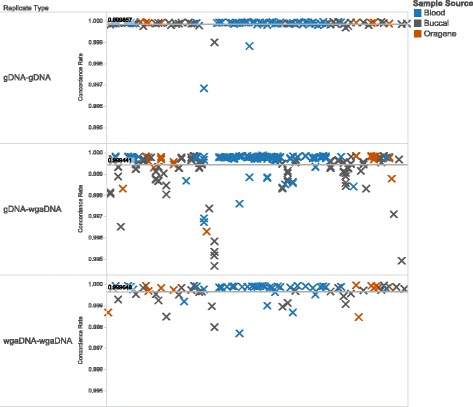
Concordance rates of replicate individuals in which both samples pass the call rate threshold of 95% by replicate type and source material: gDNA-gDNA replicates (*n* = 89), gDNA-wgaDNA replicates (*n* = 301), wgaDNA-wgaDNA replicates (*n* = 77)

Intra-individual concordance within each type of replicate comparison is shown in Fig. 4. The mean concordance rate for passing samples for gDNA replicates (*n* = 89) was 99.99% with three outliers observed. Between gDNA-wgaDNA replicates (*n* = 301), the mean concordance rate was 99.94% and between wgaDNA replicates (*n* = 77), the mean concordance rate was 99.97%. These similar findings suggest that the WGA process does not appear to introduce an appreciable systematic bias relative to gDNA input and that wgaDNA input produced highly concordant and reproducible genotypes.

### Locus completion & concordance

**Fig. 5:**
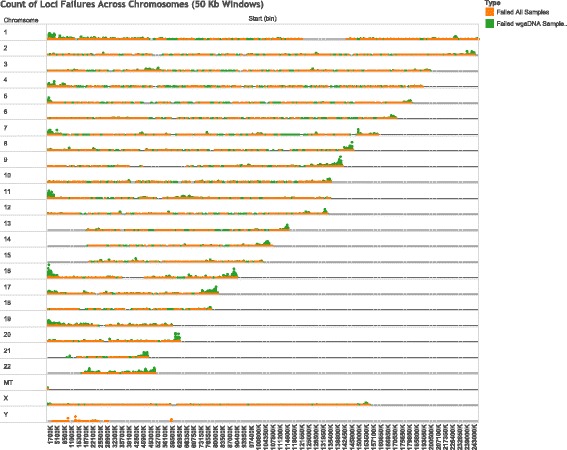
Count of failing loci within 50 kb windows for each chromosome. Loci failing all samples (gDNA and wgaDNA) in orange and loci failing only wgaDNA samples in green

Annotation of chip content was performed using the v1.1 Illumina manifest. Of the 8512 probes which failed completely, 8003 mapped to unique annotated loci, so the overall loci failure rate assigned to the array is 0.2%. Of the 50,816 probes that failed all wgaDNA samples, 48,294 were associated with a unique annotated locus, yielding an additional 1.21% locus failure rate to wgaDNA samples only. We evaluated the location of these additional failures in wgaDNA input only; they map to regions of probable under-amplification in nearly half of the chromosome ends covered by probes (Fig. 5). There does not appear to be an association, however, between low level amplification of these subtelomeric regions nor other high percentage failure regions on the chromosomes with GC-content (Additional file 1: Figure S3).

We confirmed 68,125 duplicate loci, 65,398, which were evaluated for both gDNA and wgaDNA inputs (Additional file 1: Figure S4). 98.6% of duplicate loci had greater than 99% concordance within both input types, 0.24% had lower concordance in both gDNA and wgaDNA input types, 0.07% had low concordance in gDNA samples only, and 1.6% had low concordance in wgaDNA samples only, which appear to be focused in the under-amplifying regions with large percentages of failing loci.

Of these duplicate loci compared (*n* = 65,398); 40,356 had minor allele frequency (MAF) ≤5% and 31,302 had MAF ≤1% in all individuals. The percentage of duplicate loci with less than 99% concordance was slightly higher in low MAF loci in both wgaDNA and gDNA samples (Additional file 1: Figure S5). Concordance rates in duplicate loci were not correlated to MAF of loci in either gDNA (r^2^ = .000036) or wgaDNA (r^2^ = 0.00011) input types.

### Repeat of low quality samples

Two hundred sixty-seven samples from the subsets of lower quality samples (*n* = 223 low call rate; *n* = 44 heterozygosity outliers) were repeated to assess the call and heterozygosity rates (Additional file 1: Table S2). For repeat gDNA and wgaDNA samples with the same source, call rates improved and only 2 gDNA and 4 wgaDNA samples performed below the 95% call rate threshold. For gDNA samples repeated with a wgaDNA sample, call rates improved, while 4 wgaDNA samples falling below the 95% call rate threshold, further indicating that call rates were not solely dependent upon source gDNA quality, specifically pointing more towards Infinium amplification or assay-related causes. For wgaDNA samples repeated with a different wgaDNA sample call rates improved in 123 of 129 individuals, but a large number of new wgaDNA samples still fell below the 95% call rate threshold. 72.5% of those samples were from buccal derived material all notable for high quality Identifiler results, suggesting that wgaDNA quality of buccal derived material may be more dependent upon the quality of source gDNA. These results underscore the minor but real contribution of chance and/or unforeseen laboratory deviations, suggesting repetition may capture a fraction for final analyses.

The heterozygosity rates showed differences between initial and repeat samples, though the majority of samples displaying substantive differences appeared to be associated with low call rates. When evaluating samples that were repeated due to outlying heterozygosity rates, but with call rates greater than 95%, there were still significant differences in a subset of samples, while others had little to no differences. This was seen in both gDNA samples repeated with the same gDNA sample and wgaDNA samples repeated with a new wgaDNA sample, again indicating Infinium amplification or assay-related causes.

### Implications for copy-number detection

To assess the effect of wgaDNA on copy number variant detection, we evaluated two important parameters, the Log R Ratio (LRR) and the B allele frequency (BAF). LRR is the log ratio of observed probe intensity to expected intensity; any deviations from an LRR of zero are evidence for possible copy number variation (CNV). The BAF is the proportion of hybridized sample that carries the B allele, and deviations from BAFs of 0.0, 0.5, and 1.0 are indicative of aberrant allelic representation due to sample contamination or clonal mosaicism. It is important to note that a phenomenon known as “GC waves” caused by even slight quantification differences in recommended gDNA input have been shown to decrease the signal-to-noise ratio, making detection of CNVs or other chromosomal aberrations challenging [22–25]. The standard deviations of LRR and BAF estimated by GenomeStudio™ software were compared to those estimated after applying a normalization algorithm to reduce the variation introduced by individual samples or differing input types [25] (Additional file 1: Figure S6). The standard deviation in LRR was visibly reduced by data normalization similarly for both input types. Data normalization appeared to have minimal impact on the standard deviation of BAF values. The data normalization process described does not impact genotyping calls made by GenomeStudio™, however, it is highly recommended that data normalization be performed to reduce the variability of BAF and LRR, potentially providing sufficient clarity to detect constitutional CNVs or other chromosomal abnormalities, such as detectable clonal mosaicism [24, 26]. However, wgaDNA input is still prone to inadequate data in regions notable for under-amplification or loci failure (Additional file 1: Figure S7).

We further assessed CNVs among the 102 QC replicates that had matched gDNA and wgaDNA samples (excluding *N* = 8 with call rates ≤95% and *N* = 10 with a wgaDNA sample that was too noisy for accurate CNV calling). Among these 84 individuals, 63 events were detected in 39 gDNA samples (44 gains, 18 losses, and 1 mosaic CN-LOH). In the matched wgaDNA samples for the same individuals, 57 of the same events (39 gains, 17 losses, and 1 mosaic CN-LOH) were detected (true positives), 6 events (5 gains, 1 loss) were not detected (false negatives), and no additional CNVs were detected (false positives). These results indicate a 90.5% concordance rate of CNVs between matched gDNA and wgaDNA samples.

## Discussion

In this study, we have investigated the performance metrics for wgaDNA using the Illumina technology and made a comparison with gDNA input as per commercial recommendations. Current standard laboratory protocols require a relatively large amount of DNA as starting material (~ 1500 ng), and many samples fail to qualify for study inclusion, thereby compromising the statistical power of GWAS. We sought to determine whether it was possible to use wgaDNA as input material for the high-density Illumina HumanOmni5Exome BeadChip since it was unclear whether wgaDNA would perform comparably. Our results demonstrate the value of using wgaDNA generated by the MDA technology for accurate genotyping on the high-density Illumina® Infinium® BeadChips. Furthermore, we show that the MDA wgaDNA approach can be incorporated into a high-throughput pipeline, thus enabling the possibility of substantially expanding the scope and size of genotyping studies. Others have reported on the use of whole genome amplification techniques prior to genotyping on other platforms (e.g., Affymetrix), but those studies were small in scale [4–7]. The value of our study lies in the evaluation of a large number of samples using a high-density array that includes rare or low MAF SNPs. Previous approaches have used microarrays with much lower density and fewer samples, which did not fully reflect the expected error rate of various types and qualities of source samples on extremely high-density arrays and have not evaluated the implications for copy number detection.

Notably, we observed several important technical issues related to the source material. While the failure rates for the blood- and Oragene-derived wgaDNA samples were similar, the buccal derived material had a higher failure rate. The use of the Identifiler assay performance as an indicator of the quality of wgaDNA samples represents a valuable quality assessment tool for predicting the performance of samples on the Infinium® arrays. By excluding samples that performed poorly after WGA on the basis of the Identifiler microsatellite analysis, it is plausible that low quality samples could be screened out by a technology such as Identifiler or a standard SNP screen, thereby improving the completion rate of wgaDNA input samples (Additional file 1: Figure S2). In select cases, we anticipate DNA extracted from older archived material could present a daunting challenge, but use of WGA material demonstrated that it is possible to rescue a sizable fraction of samples with low amounts of available DNA. For irreplaceable samples, it is necessary to assess the quality and size of the template as well as wgaDNA fragments using Identifiler, or an equivalent test as a screen. Since wgaDNA inputs displayed a slight increase in failure rates, compared to the gDNA input, it is likely that a slightly higher number of repeats may be necessary when utilizing such samples. However, this tradeoff is still worthwhile because alternate input types utilize substantially less original template DNA than the recommended gDNA input. Careful consideration must be applied to determine whether the cost of additional repeats obviates the improvements in sample consumption. Additionally, we found that wgaDNA samples successfully used for genotyping may still have LRR and BAF data that are too noisy to detect CNV events, even after renormalization. Additionally, although the overall concordance of CNV detection between gDNA and wgaDNA was 90.5% and no false positive CNV events were found to be introduced by WGA, we observed an increased occurrence of false negatives when using wgaDNA samples for CNV analysis. In studies that plan to use wgaDNA as an alternate input type, it is critical to analyze a sufficiently large enough set of pairs (e.g., native and wgaDNA) to provide quality assurance for assay performance and more importantly clustering analyses of both common and rare SNPs. We caution that this approach has been optimized for cell-based DNA extraction and not circulating DNA.

## Conclusion

Based on our current study, wgaDNA input is a suitable alternative to generate sufficient DNA to meet the manufacturer’s recommended DNA input for the Infinium® assay. These findings represent a major advance for molecular epidemiology studies because the ability to use lower input amounts could expand samples to include unique, rare, or precious samples with limited DNA available. In turn, this development could increase sample size and thus, improve study design and power to detect more common variants associated with disease risk [27]. Indeed, the data derived from this study enabled inclusion of substantially more samples in a recently published GWAS of breast cancer occurring among childhood cancer survivors [28]. In addition, the conservation of such samples for genotyping using this platform increases the number and type of samples for analyses in molecular studies and minimizes the impact of samples with low DNA quantities. Our results should expand the scope of future important molecular epidemiology studies designed to investigate the role of genetic variation in disease.

## Methods

### Study samples

This study includes a subset of participants from the Childhood Cancer Survivor Study (CCSS). The initial collection of buccal cell samples of survivors used a mouthwash-based approach (*n* = 1671). Later collections from the active cohort provided a saliva sample using the Oragene®·DNA Collection Kit (*n* = 3087). Peripheral-blood samples were collected from survivors with a second or subsequent neoplasm (*n* = 1149).

### Buccal

Collection of buccal cells occurred May 1999 through June 2006 using a mouthwash kit. gDNA was purified from Scope samples via Gentra Puregene Buccal Cell Kit (Qiagen) by the following methods. Sample was centrifuged for 10 min at 3200 rpm to pellet cells, supernatant discarded, and then vortexed vigorously to resuspend cells in residual supernatant. 3 mL of Cell Lysis Solution was added and vortexed for 5 s. 30 uL of Proteinase K (20 mg/mL) was added and incubated at 55 °C for 1 h. 15 uL of RNase A Solution was added to cell lysate and mixed by 25X inversion then incubated at 37 °C for 15 min to 24 h. The sample was cooled to room temp and 1 mL of Protein Precipitation Solution was added before vortexing for 20 s to mix uniformly. The sample was placed in an ice bath for 10 to 30 min then centrifuged for 10 min at 3200 rpm to pellet. Supernatant was transferred into a clean tube containing 3 mL 2-propanol and 5 uL of Glycogen Solution then mixed by 50X inversion and incubated at room temp for 5 min. Samples were centrifuged for 10 min at 3200 rpm and supernatant removed. A wash was performed by adding 3 mL of 70% Ethanol, swirled several times and centrifuged for 5 min at 3200 rpm. Ethanol was carefully poured off and sample allowed to air dry for 15 min. 200 to 4000 uL of DNA Hydration solution was added, depending on pellet size, to rehydrate the DNA and incubated at 65 °C for 1 h or overnight. DNA samples were divided equally between three tubes and then stored at − 80 °C.

### Oragene

Collection of saliva sample using the Oragene®·DNA Self-Collection Kit (DNA Genotek) occurred December 2007 through 2012. gDNA was purified from Oragene®·DNA/saliva sample via manual procedures according to manufacturer’s protocol. Samples were incubated at 50 °C in a water incubator for a minimum of 1 h or in an air incubator for a minimum of 2 h. 500 uL of the mixed Oragene®·DNA/saliva sample was transferred to a 1.5 mL microcentrifuge tube. 20 uL of Oragene®·DNA Purifier was added to the tube and mixed by vortexing for a few seconds. The sample was incubated on ice for 10 min then centrifuged at room temperature for 10 min at 4000 rpm. The clear supernatant was carefully transferred, by pipet, into a fresh tube. 500 uL of 95–100% ethanol was added to 500 uL of supernatant and gently mixed by 10× inversion. The sample was incubated for 10 min at room temperature to allow the DNA to fully precipitate. The tube was then centrifuged for 10 min at 4000 rpm. The supernatant was then removed by pipet and discarded. Wash was performed by adding 1 mL of 70% Ethanol and centrifuged for 5 min at 4000 rpm. Ethanol was carefully removed, by pipet, and sample allowed to air dry for 20 min. 1 mL of Buffer AE (Qiagen) was added to rehydrate the DNA and incubated at 50 °C for 1–2 h. DNA samples were divided equally and then stored at − 80 °C.

### Blood

Collection of blood samples occurred 2001 through 2012. gDNA was purified from neutrophils via Gentra Puregene Kit (Qiagen) according to the following method. 15 mL RBC lysis solution was added to neutrophil portion of sample and inverted several times, incubated for 5 to 10 min at room temperature until clear red. Sample was centrifuged for 5 min at 1500 rpm to pellet. Supernatant was discarded and cells were transferred to 1 mL of 1X PBS, centrifuged for 4 min at 14000 rpm to pellet. Supernatant was discarded and 100 uL of 1X PBS added to cells and vortexed to mix. 300 uL of Cell Lysis Solution was added and vortexed to mix. 1.5 uL of RNase Solution was added and vortexed to mix, sample was incubated at 37 °C for 5 min. 20 uL Proteinase K was added and vortexed to mix, sample was incubated at 50 °C for 1 h. 100 uL of Protein Precipitation Solution was added and vortexed to mix, sample was incubated on ice for 5 min. Sample was centrifuged for 5 min at 14000 rpm to pellet. Supernatant was transferred to a fresh tube containing 300 uL 2-propanol and gently mixed by 50× inversion. Sample was centrifuged for 5 min at 14000 rpm to pellet DNA. Supernatant was removed by pipet and discarded. Wash was performed by adding 300 uL of 70% Ethanol and centrifuged for 1 min at 14000 rpm. Ethanol was carefully removed, by pipet, and sample allowed to air dry for 20 min. 50 to 100 uL of DNA Hydration solution was added, depending on pellet size, to rehydrate the DNA and incubated at 50 °C for 1 h and then overnight at room temp. DNA samples were then stored at − 80 °C.

### Quantification

Both gDNA and wgaDNA samples were quantified using the Quant-iT™ PicoGreen® dsDNA Reagent (Life Technologies) according to manufacturer’s protocol. All samples were normalized to desired concentration for input into Illumina® Infinium® LCG assay which is 50 ng/μL.

### Whole genome amplification

Target input of 50 ng* of each gDNA sample (as determined by PicoGreen®) was used as template for the illustra™ GenomiPhi™ V2 DNA Amplification Kit (GE Healthcare), performed exactly according to the manufacturer’s protocol. Samples were denatured by heat and then isothermally amplified using φ29 DNA Polymerase at 30 °C for 1 h and 30 min. For those samples in which an extended amplification time was used, isothermal amplification at 30 °C was 3 h. *To utilize material from precious samples, less than 50 ng of input was used successfully in some samples.

### Identifiler

2 ng of each gDNA and wgaDNA sample (as determined by PicoGreen®) was used as template for the AmpF*l*STR® Identifiler® assay (Life Technologies) according to manufacturer’s protocol. Alleles were scored using GeneMapper 4.0 software with a peak height threshold of 200 RFUs. Genotypes for gDNA samples were compared to wgaDNA samples at all 16 markers and were evaluated for contamination, completion and concordance.

### Illumina® Infinium® LCG assay

High-throughput, genome-wide SNP genotyping, using Infinium® HumanOmni5Exome + BeadChip technology (Illumina, Inc.), was performed at the Cancer Genomics Research Laboratory (CGR) of the National Cancer Institute. Genotyping was performed according to manufacturer’s guidelines using the Infinium® LCG Quad Assay automated protocol using 400 ng of input amount for both gDNA and wgaDNA. Samples were denatured and neutralized then isothermally amplified by whole-genome amplification. The amplified product was enzymatically fragmented, then precipitated and re-suspended before hybridization to the BeadChip. Single-base extension of the oligos on the BeadChip, using the captured DNA as a template, incorporated detectable labels on the BeadChip, and determined the genotype call for the sample. The Illumina® iScan™ scanned the BeadChips at two wavelengths to create image files.

### Data analysis

The image files containing fluorescence signals were imported into the commercial genotype analysis software, GenomeStudio™ (Illumina, Inc.). This software is used to compute the two principal types of data for assayed SNP loci, affinity-normalized probe intensities and genotypes. Illumina’s GenCall™ genotype calling method within the software was used to cluster the initial 6258 samples and estimate genotypes. Samples were clustered separately for gDNA and wgaDNA samples. This decision was based on previous testing of small size pilot data sets which demonstrated differential clustering at some loci between gDNA and wgaDNA input types, when clustering by input type greater concordance was seen between the input types. Clustering for both groups was performed using the self-clustering method, using available samples to calibrate the cluster positions to the data. A no-call threshold, or GenCall™ (GC) score, of 0.25 was used to determine the call rate for each sample during clustering. This GenCall™ score is a confidence measure assigned to each call that is used to filter poor quality calls, SNPs, or samples. Data on called genotype, genotype call quality score; genotype raw and normalized probe intensities, Log R ratio, and B allele frequency for each assay were exported from GenomeStudio™ using their Genotype Final Report (GRF) format. Using GFR as input, the high-performance binary file format (GDAT) was generated.

### Data normalization and copy number variation

The LRR value for each SNP or CNV assay provided data on probe intensity relative to that of the estimated genotype-specific cluster location. Information on allelic ratio was provided by BAF, which is derived from the ratio of probe values relative to the locations of the estimated genotype-specific cluster locations. LRR and BAF were estimated by the GenomeStudio™ software, but these estimates can suffer from bias due to the properties of the assay chemistry and fluorescent dyes used in the probes and concentration of DNA input, which can reduce precision in estimating copy-number and allelic imbalances. A data normalization method was implemented to re-estimate LRR and BAF after applying quantile normalization with an enhanced multiple regression model, incorporating within-chip signal rescaling terms and a polynomial correction for GC and CpG waves was performed as described using GLU software package (http://code.google.com/p/glu-genetics/) [24]. The renormalized LRR and BAF values were then analyzed using custom software pipelines that involved BAF Segmentation packages (http://baseplugins.thep.lu.se/wiki/se.lu.onk.BAFsegmentation) with circular binary segmentation algorithm to detect copy number variation with minimum of 20 probes per segment to minimize the false discovery. All potential events were plotted and false positives were excluded from analysis based on manual review.

## Additional file

Additional file 1: Supplementary Tables and Figures. (DOCX 898 kb)

## Abbreviations

BAF: B allele frequency
CN-LOH: copy neutral-loss of heterozygosity
CNV: copy number variation
DNA: deoxyribonucleic acid
gDNA: genomic DNA
GWAS: Genome-wide association study
LRR: Log R ratio
MAF: minor allele frequency
MDA: multiple displacement amplification
QC: quality control
SNP: single nucleotide polymorphism
WGA: whole genome amplification
wgaDNA: whole genome amplified DNA
