# Supplementary material for: Successful use of whole genome amplified DNA from multiple source types for high-density Illumina SNP microarrays
Source: BMC Genomics. 2018 Mar 6;19:182. doi: 10.1186/s12864-018-4572-6 (PMC5838969; doi:10.1186/s12864-018-4572-6)

**Additional File 1: Supplementary Tables and Figures**

***Table 1.*** *Number of samples with call rates below 95% by chip.*

| **Number of Failing Samples on Chip** | **Number of Chips** |
| --- | --- |
| **0** | 1178 |
| **1** | 280 |
| **2** | 61 |
| **3** | 16 |
| **4** | 8 |

***Figure 1****. Call rates of replicate samples by input type and replicate type. gDNA replicates in blue, wgaDNA replicates in orange, and gDNA/wgaDNA replicates in red.*

***Figure 2****. a) Initial failure rates for wgaDNA input types by source material (blood, buccal, Oragene). b) Classification of quality of wgaDNA input, based on Identifiler assay results, by source material c) Hypothetical reduced call rates for wgaDNA input by source material if poor quality wgaDNA samples would have been excluded from project.*


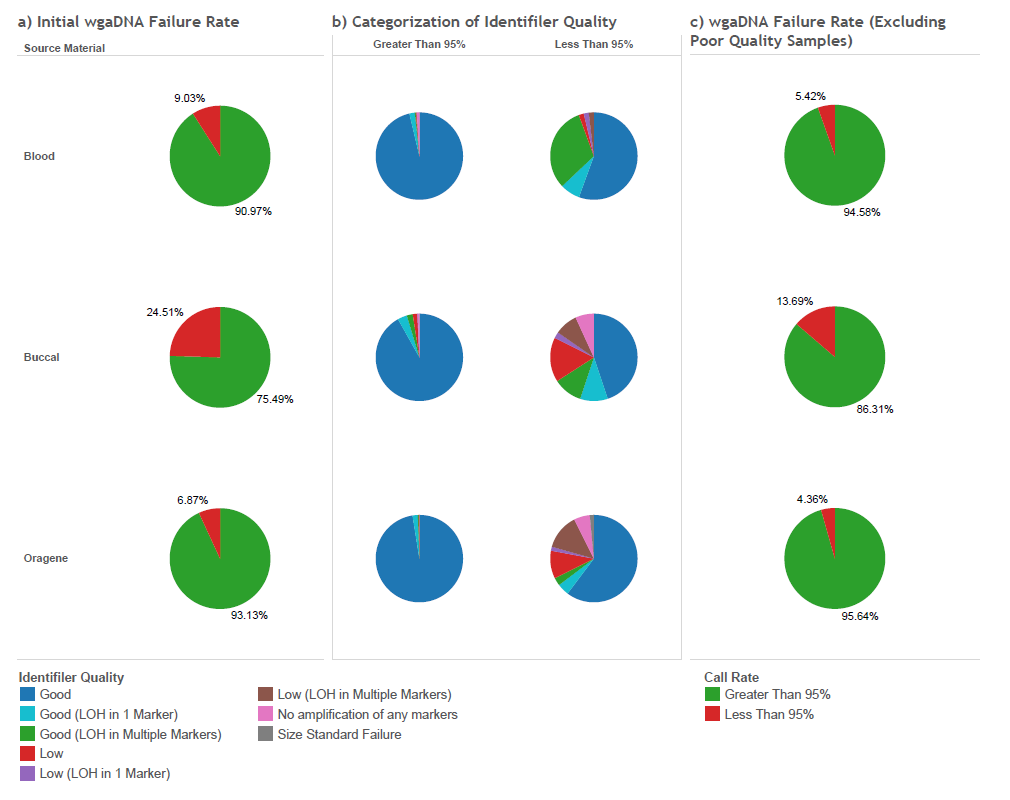


***Figure 3****. a) Percentage of failing loci within 50 kb windows for chromosomes 1, 5 and 7. Percentage of loci failing all samples (gDNA and wgaDNA) on negative Y-axis and percentage of loci failing only wgaDNA samples on positive Y-axis. b) Plots of GC content to percentage of failing loci for each input type showing now correlation (gDNA: r^2^= X, wgaDNA: r^2^=X) within 50 kb windows. GC content within window is defined by color, where dark blue is lower GC content and dark orange is higher GC content.*

***Figure 4****. Concordance of duplicate loci (n=65,592) by input type (gDNA vs. wgaDNA). Color depicts loci that fall above (green) or below (red) the concordance rate threshold of 99%, which is displayed on each axis as a dotted line.*

***Figure 5.*** *Distribution of MAF in duplicate loci. Inset: Percentage of duplicate loci with less than 99% concordance in both gDNA (top) and wgaDNA (bottom).*

***Table 2.*** *Pass/Fail results of repeated samples. Samples were repeated due to either low call rate (below threshold of 95%) or outside the expected range for heterozygosity rate (11% to 14%). Individuals were repeated with the same sample (gDNA or wgaDNA), a wgaDNA sample if not enough gDNA was remaining, or a new wgaDNA sample if not enough wgaDNA was remaining. The number of samples which then passed or failed each threshold is provided, indicating that gDNA quality and wgaDNA quality are not the only contributing factors to samples*

| **Repeat Reason** | **Input Type** | **Repeat Input Type** | **Pass Call Rate Threshold**  **>0.95** | **Fail Call Rate Threshold**  **<0.95** | **Pass Heterozygosity Threshold**  **0.11-0.14** | **Fail Heterozygosity Threshold**  **<0.11 or >0.14** |
| --- | --- | --- | --- | --- | --- | --- |
| **Low Call Rate (n=223)** | |  |  |  |  |  |
|  | gDNA (n=90) | Same gDNA Sample (n=44) | 42 | 2 | 39 | 5 |
|  |  | wgaDNA Sample (n=46) | 42 | 4 | 43 | 3 |
|  | wgaDNA (n=133) | Same wgaDNA sample (n=41) | 37 | 4 | 37 | 4 |
|  |  | New wgaDNA sample (n=92) | 58 | 34 | 69 | 23 |
| **Heterozygosity Outlier (n=44)** | |  |  |  |  |  |
|  | gDNA (n=7) | Same gDNA Sample (n=6) | 6 |  | 4 | 2 |
|  |  | wgaDNA Sample (n=1) | 1 |  | 1 |  |
|  | wgaDNA (n=37) | New wgaDNA sample (n=37) | 31 | 6 | 34 | 3 |

***Figure 6.*** *Standard deviation raw (left) vs. normalization (right) of LRR (top) and BAF (bottom) by input type (gDNA, wgaDNA) within samples (n=242) from replicate individuals.*


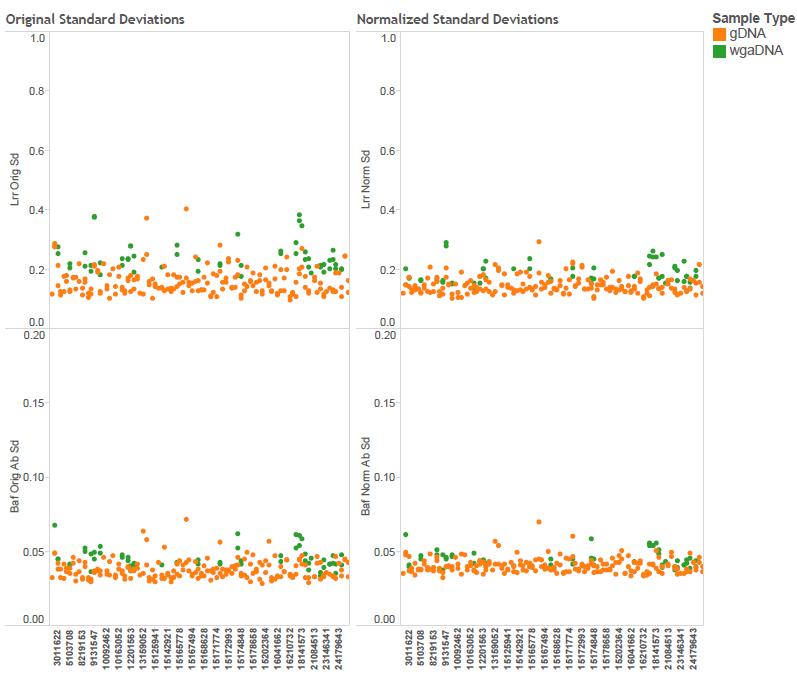


***Figure 7.***  *Plots of raw LRR and BAF across Chromosome 20 for gDNA and wgaDNA samples of the same individual. The data from the wgaDNA sample is slightly noisier, when compared to the gDNA sample, especially at the distal q arm, which was a region with large numbers of entire loci failure within wgaDNA samples (see Figure 1). But the CNV on the p arm, where loci completion was similar between the two input types, can be seen in both instances.*


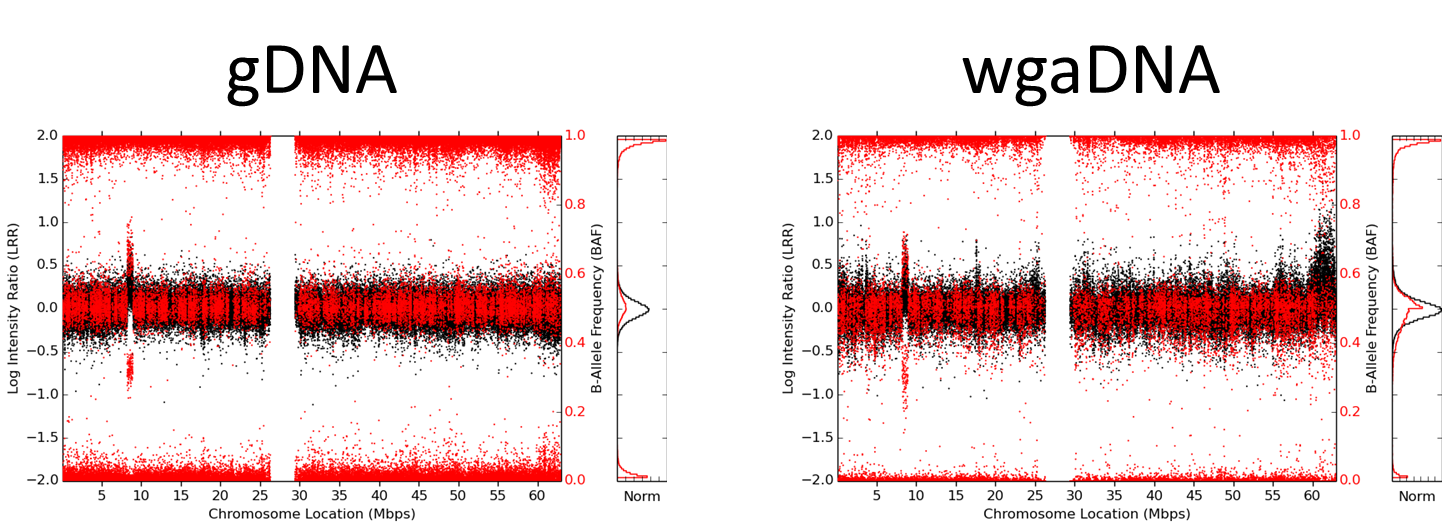

Supplement: Supplementary file 1 — Supplementary Tables and Figures. (DOCX 898 kb) [file 12864_2018_4572_MOESM1_ESM.docx]
